# Novel Mutation m.10372A>G in *MT-ND3* Causing Sensorimotor Axonal Polyneuropathy

**DOI:** 10.1212/NXG.0000000000000566

**Published:** 2021-03-15

**Authors:** Helene Bruhn, Kristin Samuelsson, Florian A. Schober, Martin Engvall, Nicole Lesko, Rolf Wibom, Inger Nennesmo, Javier Calvo-Garrido, Rayomand Press, Henrik Stranneheim, Christoph Freyer, Anna Wedell, Anna Wredenberg

**Affiliations:** From the Department of Medical Biochemistry and Biophysics (H.B., R.W., C.F., A. Wredenberg), Karolinska Institutet; Centre for Inherited Metabolic Diseases (H.B., R.W., C.F., M.E., N.L., H.S., A. Wedell, A. Wredenberg), Karolinska University Hospital; Department of Clinical Neuroscience (K.S., R.P.), Karolinska Institutet; Department of Neurology (K.S., R.P.), Karolinska University Hospital; Department of Molecular Medicine and Surgery (F.A.S., M.E., N.L., J.C.-G., H.S., A. Wedell), Karolinska Institutet; Department of Pathology (I.N.), Karolinska University Hospital; and Science for Life Laboratory (H.S.), Karolinska Institutet, Stockholm, Sweden.

## Abstract

**Objective:**

To investigate the pathogenicity of a novel *MT-ND3* mutation identified in a patient with adult-onset sensorimotor axonal polyneuropathy and report the clinical, morphologic, and biochemical findings.

**Methods:**

Clinical assessments and morphologic and biochemical investigations of skeletal muscle and cultured myoblasts from the patient were performed. Whole-genome sequencing (WGS) of DNA from skeletal muscle and Sanger sequencing of mitochondrial DNA (mtDNA) from both skeletal muscle and cultured myoblasts were performed. Heteroplasmic levels of mutated mtDNA in different tissues were quantified by last-cycle hot PCR.

**Results:**

Muscle showed ragged red fibers, paracrystalline inclusions, a significant reduction in complex I (CI) respiratory chain (RC) activity, and decreased adenosine triphosphate (ATP) production for all substrates used by CI. Sanger sequencing of DNA from skeletal muscle detected a unique previously unreported heteroplasmic mutation in mtDNA encoded *MT-ND3*, coding for a subunit in CI. WGS confirmed the mtDNA mutation but did not detect any other mutation explaining the disease. Cultured myoblasts, however, did not carry the mutation, and RC activity measurements in myoblasts were normal.

**Conclusions:**

We report a case with adult-onset sensorimotor axonal polyneuropathy caused by a novel mtDNA mutation in *MT-ND3*. Loss of heteroplasmy in blood, cultured fibroblasts and myoblasts from the patient, and normal measurement of RC activity of the myoblasts support pathogenicity of the mutation. These findings highlight the importance of mitochondrial investigations in patients presenting with seemingly idiopathic polyneuropathy, especially if muscle also is affected.

Around a third of patients with mitochondrial disorders develop peripheral neuropathy, but this is mostly mild or subclinical.^1^ However, mutations in genes involved in mitochondrial dynamics (*MFN2, GDAP1*),^2–6^ in mtDNA replication and maintenance (*POLG, TWNK, TYMP, MPV17*)^7–10^ or RC complex V *MT-ATP6,*^11–14^ are known to cause peripheral neuropathy as the initial manifestation or dominant feature of mitochondrial disease. Some mutations in mtDNA encoded RC complex I (CI) subunits, *MT-ND1*, *MT-ND4*, and *MT-ND6* associated with Leber hereditary optic neuropathy, are known to have neuropathy as a minor feature of disease.^1,15,16^ To date, known pathogenic mutations in *MT-ND3* are only described in patients with Leigh syndrome with or without dystonia, in mitochondrial encephalomyopathy, lactic acidosis, and stroke-like episodes, or in patients comprising with overlapping clinical features from both syndromes.^17–20^

If an inherited neuropathy is due to mitochondrial disease, the patients need to be thoroughly investigated for involvement of other organs or systems. Treatment with cofactors and antioxidants such as coenzyme Q10 (ubiquinone), ascorbic acid, vitamin E, lipoic acid, riboflavin, thiamin, niacin, vitamin K, creatine, and carnitine has been suggested,^21,22^ and engagement of other organs may demand specific therapy.

In this study, we describe a female patient with onset of neuropathy symptoms due to a small- and large-fiber sensorimotor axonal polyneuropathy at age 41 years, as the initial manifestation. During the following years, she also developed a myopathy and experienced fluctuating bilateral blurred vision. The disease was shown to be caused by a novel mutation in *MT-ND3*, which was found at high levels of heteroplasmy in DNA from muscle tissue. In contrast, the mutation was not detectable in blood, urinary epithelial cells, or cultured myoblasts and found only at very low levels in cultured fibroblasts. Our finding emphasizes the importance of mitochondrial investigation including muscle biopsy in patients with polyneuropathy of unknown genetic cause, especially when coexisting muscular symptoms occur.

## Methods

### Clinical Evaluation

See supplementary material (links.lww.com/NXG/A388).

### Muscle and Skin Biopsy Samples and Establishment of Cell Cultures

See supplementary material (links.lww.com/NXG/A388).

### Biochemical Investigations of Skeletal Muscle and Cultured Myoblasts

ATP production rate and RC enzyme activities were measured in isolated mitochondria from both muscle biopsies as described earlier.^23^ For determination of RC enzyme activities in cultured myoblasts, mitochondria were isolated by homogenization of cells in a homogenizing and washing buffer (320 mM sucrose, 1 mM ethylene glycol tetraacetic acid, and 20 mM Tris-HCl pH7.2 with 0.1% bovine serum albumin [BSA]), followed by differential centrifugation. The final mitochondrial pellet was resuspended in resuspension buffer (250 mM sucrose, 15 mM K_2_HPO_4_, 2 mM MgAc_2_, 0.5 mM EDTA, and 0.5 g/L BSA, pH 7.2) before determination of RC enzyme activities as previously described.^23^ The rate of mitochondrial ATP production was determined in permeabilized myoblasts. The permeabilization procedure was performed on an ice bath and using a refrigerated centrifuge. Two solutions were used; A: 0.1 g/L digitonin, 150 mM KCl, 25 mM Tris, 10 mM K_2_HPO_4_, 2 mM EDTA and 1 g/L BSA, pH 7.5); B: same as A but with digitonin omitted. Approximately 1 million myoblasts at a confluency of 80-90% were harvested for each experiment. The cell pellet was incubated for 10 minutes with 100 μL of solution A. After incubation cells were centrifuged for 3 minutes at 700*g* and washed twice with 100 μL of solution B. Finally, cells were resuspended in 100 μL of solution B. ATP production was measured in white 96-well microtiter plates. To each well, ATP monitoring reagent, adenosine diphosphate, and one of the following substrate combinations were added: glutamate + malate, pyruvate + malate, or succinate + rotenone. Each substrate combination was measured in the absence or presence of oligomycin (OM). Reaction was started by addition of approximately 1 × 10^3^ cells/well, and the increase in light emission was followed for 10 minutes at 25°C in a Perkin Elmer Victor^2^ plate reader. Measurements were standardized by determining the subsequent increase in light emission after addition of 500 nM ATP to wells containing all the above-described combinations of substrates and OM, but without cells. Each measurement was repeated in 4 wells. In total, 24 wells were used per sample investigated, and 24 wells were used for the calibration procedure. Mitochondrial ATP production in myoblasts was calculated as *ATP production rate (−OM) – ATP production rate (+OM)* for each substrate combination. Final reagent concentrations and principles of the measurement procedure were as previously described.^24^

### Morphologic Investigation of Skeletal Muscle

See supplementary material (links.lww.com/NXG/A388).

### Genetic Analysis

See supplementary material (links.lww.com/NXG/A388).

### Blue Native Polyacrylamide Gel Electrophoresis and In Silico Analyses

See supplementary material (links.lww.com/NXG/A388).

### Standard Protocol Approvals, Registrations, and Patient Consents

This study has been approved by the regional ethical board in Stockholm, Sweden (2014/995-32). Written informed consent was obtained from the patient involved.

### Data Availability

The data that support the findings of this study are available from the corresponding author on reasonable request.

## Results

### Clinical History

A 46-year-old woman with no family history of neuromuscular disease or other clinical symptoms typically associated with mitochondrial diseases presented with a 5-year history of painful paresthesia and numbness, which started in her toes but later progressed to her feet and hands. During the same period, she also experienced fluctuating muscular weakness in the lower and upper extremities. The initial neurologic examination showed normal walking capacity and a minor distal weakness in both upper and lower extremities, but no fasciculations in the extremities. The patient had reduced light touch sensibility in the lower legs, reduced pinprick sensibility in the feet, and lack of vibration sensibility in the ankles. The reflexes were generally reduced, with a total loss of ankle reflexes. The patient had no ataxia, cranial nerve affection, or Babinski sign. Nerve conduction studies and quantitative sensory testing showed a symmetric sensorimotor axonal polyneuropathy as well as signs of small fiber involvement (table e-1, links.lww.com/NXG/A388). The EMG showed a mostly chronic neurogenic pattern and no signs of myopathy (table e-2). To screen for known etiologic causes of her sensory-predominant neuropathy we performed a comprehensive work-up. The patient denied alcohol overconsumption. Routine basal blood samples including serum cobalamin, folate acid and homocysteine, oral glucose tolerance test and serum protein electrophoresis were normal. Antibodies for rheumatic disease (antinuclear antibodies, rheumatoid factor and anti-neutrophils cytoplasmic antibodies) were negative. Screening for hepatitis C, HIV, Lyme disease and syphilis was also negative. Transglutaminase antibodies (Celiac disease), abdominal fat biopsy and *TTR* gene sequencing (Amyloidosis) were normal. Urine trihexoside was normal and *GLA* gene sequencing (Fabry disease) did not reveal any disease-causing variants. To identify a possible inflammatory etiology, a muscle biopsy with a strict morphologic analysis was performed. Surprisingly, the muscle biopsy showed ragged red fibers indicating a mitochondrial disorder, but no signs of myositis or vasculitis. To expand the mitochondrial investigation, the patient was referred to the Centre for Inherited Metabolic Diseases for further investigation, as described below. During the following year, the patient experienced a gradual increase in the level of muscle weakness leading to inability to walk without using a walking aid. A follow-up EMG at this time revealed a generalized myopathic pattern (table e-2). Blood workup including creatine kinase levels was normal. During the following 3 years, the patient also experienced fluctuating bilateral blurred vision. The eye examination showed punctate retinal hemorrhages of unknown etiology in the right eye and no optic disc changes, although visual evoked potential revealed bilateral prolonged latencies indicating optic nerve affection, which was assumed to be related to her mitochondrial condition. Since then, repeated eye assessments have been performed. The patient has a Leber miliary aneurysm in the right retina and has received laser treatment. She has also undergone bilateral cataract surgery as well as epiretinal membrane removal and vitrectomy at her right eye. None of these conditions are considered to be associated with her mitochondrial disorder. At the time of diagnosis, investigation of other organ systems included normal echocardiography and an auditory test. MRI of the brain showed a slight global parenchymal atrophy, but no white matter changes. A repeated MRI of the brain at age 58 years showed similar results. The proximal muscle weakness has worsened during the years leading to the use of a power wheelchair outdoors at age 58 years, although the patient is still capable of walking short distances without aid indoors. She complains of muscle soreness, clumsiness in her hands, and fatigue, which worsen with physical activity. She has tried coenzyme Q10 and creatinine without effect. She has continuous severe neuropathic pain and uses gabapentin and sometimes tramadol. Earlier use of tricyclic antidepressant and duloxetine had no effect or troublesome side effects.

### Biochemical and Morphologic Investigations Show Clear Evidence of Mitochondrial Dysfunction

**Figure 1:**
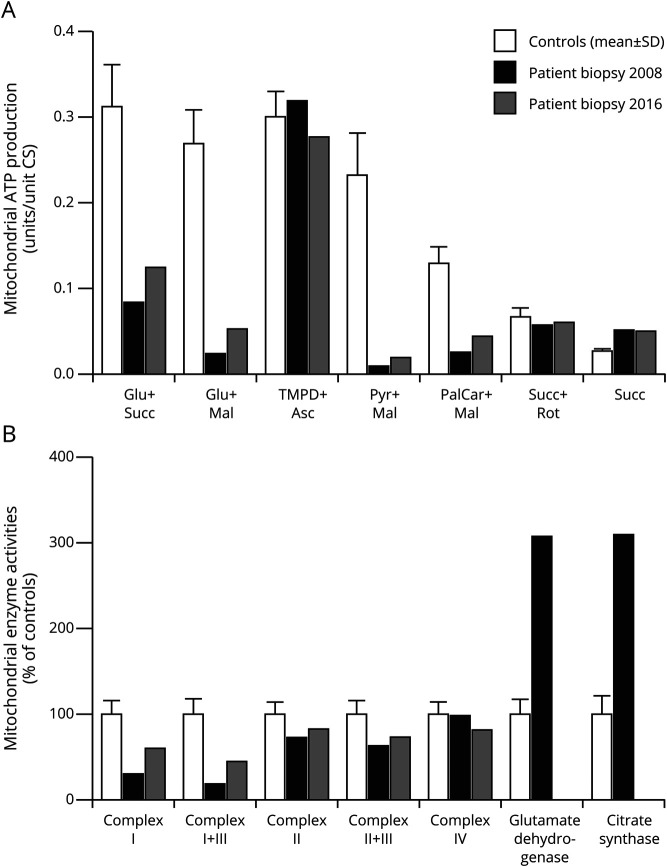
Biochemical Investigations of Mitochondria From Skeletal Muscle Biopsies in 2008 and 2016 Show Complex I (CI) Deficiency (A) Mitochondrial ATP production (MAPR) is decreased with all substrates involving CI. Only TMPD + ascorbate (complex IV) and succinate + rotenone (complex II + III + IV) show normal activities. (B) CI and CI + complex III in the respiratory chain show markedly decreased activities. Complex II and II + III are slightly reduced while the activity for complex IV is normal. The activities of the mitochondrial control enzymes glutamate dehydrogenase and citrate synthase are threefold increased compared with the controls in biopsy from 2008 (not measured 2016), indicating mitochondrial proliferation in the muscle tissue. The control group for MAPR consisted of 18 individuals aged 7–70 years. For the enzyme activity determinations, several control groups were used, consisting of 10–119 individuals aged 5–80 years. All controls were thoroughly chosen among patients referred to the clinic for a muscle biopsy. Inclusion criteria were normal morphologic analysis of muscle, no pathogenic findings in genetic analyses, and a clinical picture not suggesting mitochondrial disease. Results diverging more than ±2 SDs from the average of the control groups were considered pathologic.

**Figure 2:**
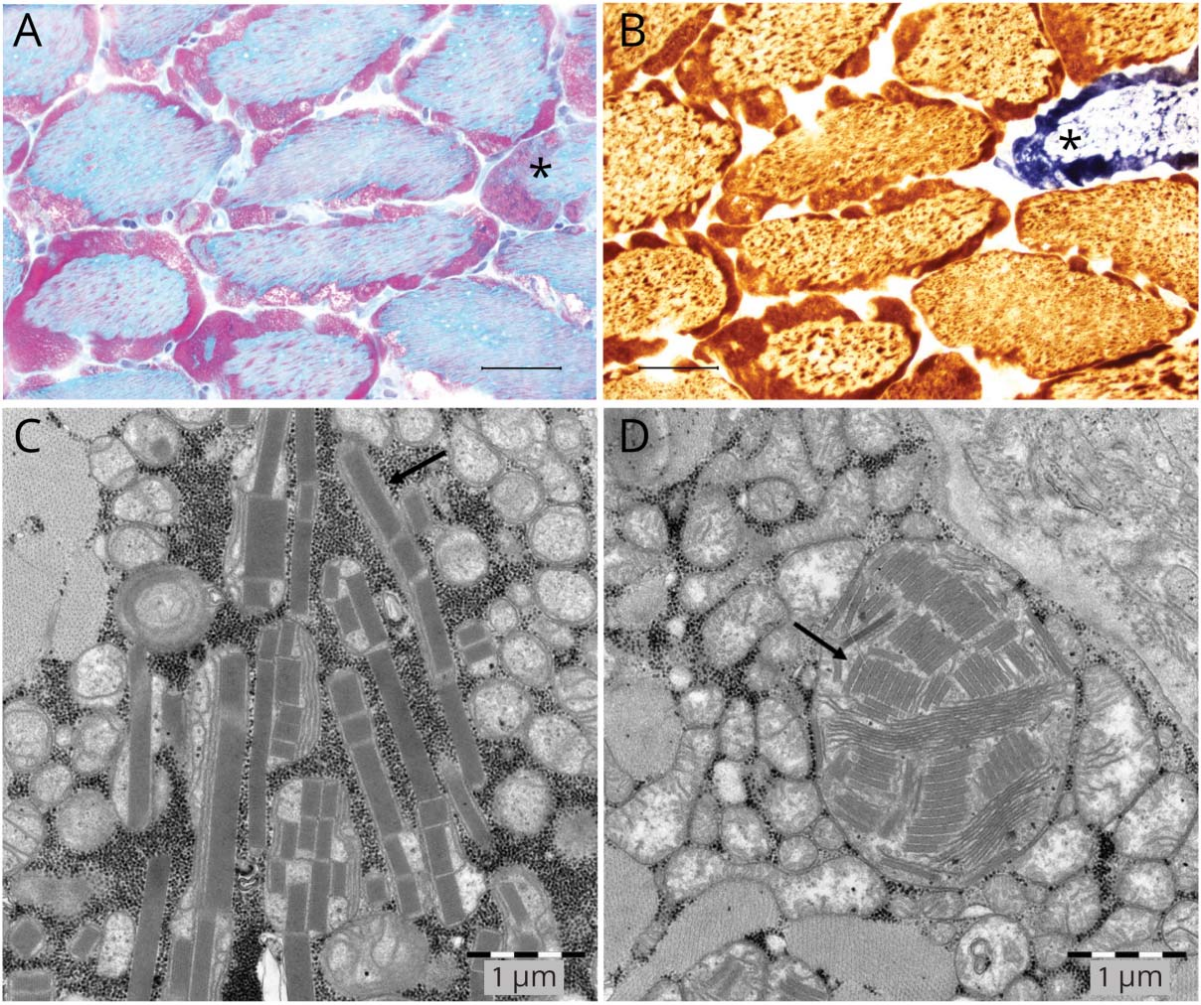
Morphologic Analysis and Electron Microscopy of Skeletal Muscle Showing Clear Evidence of Mitochondrial Disease (A) Gomori trichrome staining shows numerous ragged red fibers, bar 50 μm (B), which are shown to be COX positive in the combined cytochrome oxidase/succinate dehydrogenase staining except for 1 fiber, which is COX negative (marked with*), bar 50 μm. (C + D) Electron microscopy shows abnormal mitochondria with paracrystalline inclusions (marked with arrows).

A muscle biopsy for mitochondrial investigation was conducted in 2008 when the patient was aged 47 years. Measurement of mitochondrial ATP production and the RC activity in mitochondria isolated from muscle was performed. Mitochondrial ATP production was decreased using all substrates involving CI. Only N,N,N',N'-Tetramethyl-p-phenylenediamine (TMPD) + ascorbate (complex IV) and succinate + rotenone (complex II + III + IV) showed normal activities (figure 1A). RC activity showed markedly decreased activities for CI and CI + complex III. Also, complex II and II + III were slightly reduced while the activity for complex IV was normal (figure 1B). The activities of the mitochondrial control enzymes glutamate dehydrogenase and citrate synthase were threefold increased compared with the controls, indicating mitochondrial proliferation in the muscle tissue (figure 1B). Morphologic analysis was performed on muscle tissue from the same biopsy. Gomori trichrome staining revealed numerous ragged red fibers (RRFs) indicating accumulation of abnormal mitochondria below the plasma membrane of the muscle fiber (figure 2A). All fibers, except 1 COX-negative fiber, were shown to be COX positive in cytochrome oxidase staining indicating normal complex IV function of the mitochondria (figure 2B). Electron microscopy demonstrated abnormal collection of mitochondria with paracrystalline inclusions (figure 2, C and D).

### Sanger Sequencing Identified a Novel Mutation m.10372A>G in Muscle Tissue

To search for the genetic cause of the disease, mtDNA isolated from muscle was analyzed by Sanger sequencing. The analysis detected a novel heteroplasmic mutation in *MT-ND3*, m.10372A>G (predicting p.(Glu105Gly)). *MT-ND3* is one of the mtDNA-encoded subunits of CI in the RC. The identified mutation had a high heteroplasmic level in muscle according to the Sanger electropherogram, almost no wild-type mtDNA was detected (not shown). Sanger sequencing could not detect the mutation in DNA from blood, cultured fibroblasts, or urinary epithelial cells from the patient.

### Quantification by Last-Cycle Hot PCR Showed High Levels of Mutated mtDNA in Muscle but Not in Other Tissues

**Figure 3:**
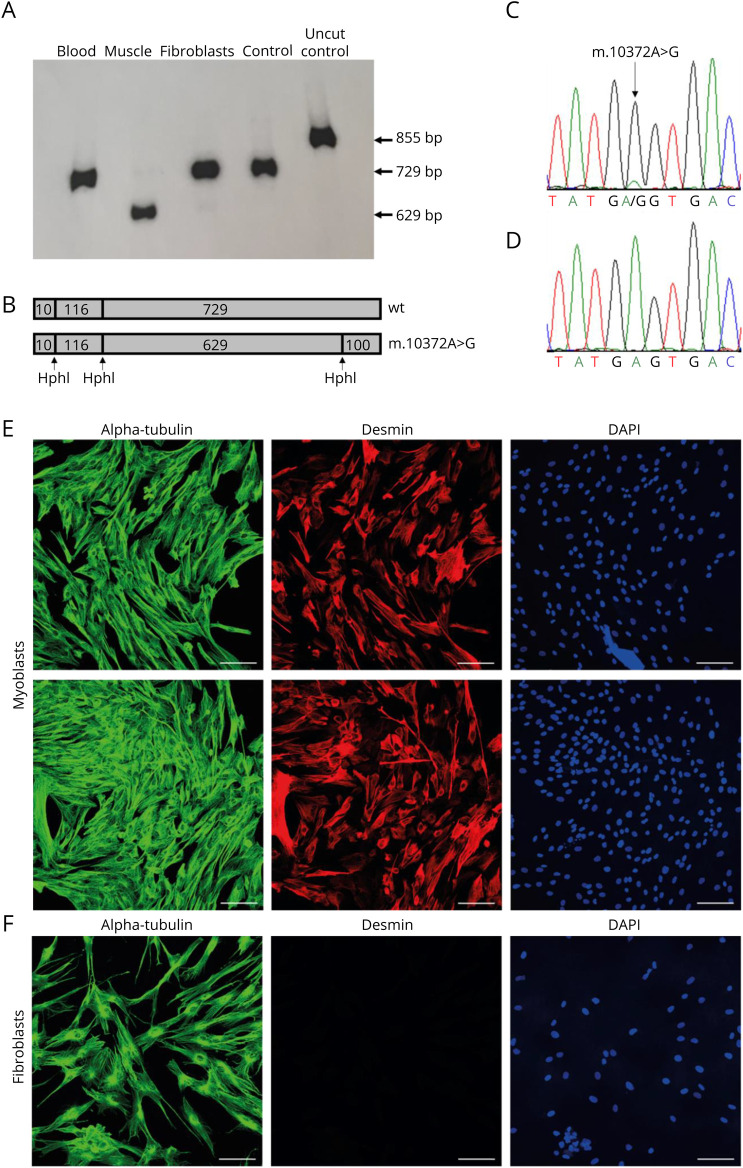
Last-Cycle-Hot PCR (LCH-PCR), Sanger Sequencing, and Immunocytochemistry (A) LCH-PCR analysis. *HphI* digested mitochondrial DNA (mtDNA) in blood from the patient (lane 1), skeletal muscle from the patient biopsy from 2008 (lane 2), cultured fibroblasts from the patient (lane 3), wild-type control (lane 4), and undigested wild-type control DNA (lane 5). The mutation is heteroplasmic, and quantification shows heteroplasmy of 97% in muscle and 3% in fibroblasts, but none in blood. (B) The expected fragment sizes after *HphI* digestion of normal and mutated mtDNA are shown. (C) Sanger sequencing of mtDNA from skeletal muscle from patient biopsy from 2016 shows high heteroplasmic level of m.10372A>G. (D) Sanger sequencing of mtDNA from myoblasts from 2016 biopsy cannot detect the mutation. (E + F) Patient's myoblasts and fibroblasts immunostained for alpha-tubulin (green) and myogenic marker desmin (red). Nuclei counterstained with 4',6-diamidino-2-phenylindole (blue). Scale bar = 100 μm.

To further characterize the mutation, mtDNA from blood, cultured fibroblasts, and muscle was quantified by last-cycle hot PCR. Quantification showed 97% mutated mtDNA in muscle and 3% in fibroblasts, but undetectable in blood (figure 3, A and B).

### Blue Native Polyacrylamide Gel Electrophoresis Analysis of Muscle Mitochondria Resulted in Normal Complex I Assembly

**Figure 4:**
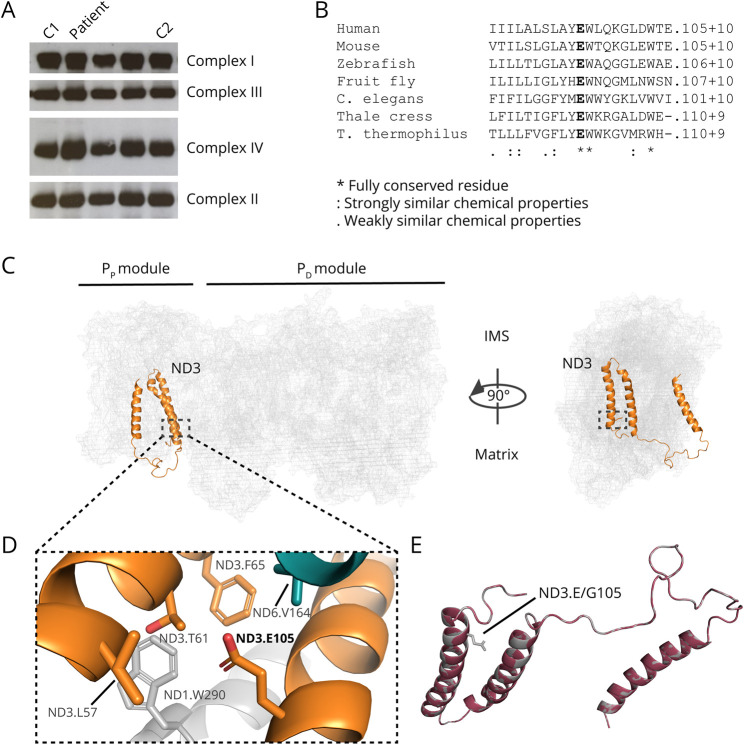
Blue Native Polyacrylamide Gel Electrophoresis (BN-PAGE) and In Silico Analyses (A) BN-PAGE of mitochondria isolated from skeletal muscle from the patient and controls (C1 and C2). No deviation in the quantity or size of complex I (CI) in the patient was seen compared with controls, indicating that the mutation in *MT-ND3* does not affect assembly of CI. Lanes 3 and 4 represent patients not included in this study. (B) Clustal omega alignment of different species shows that the residue E105 is highly conserved down to the *T thermophilus* homolog *Nqo1*. (C) Location of ND3 in a human CI structure (PDB: 5XTC) seen from 2 different angles, relative to the mitochondrial matrix and intermembrane space. E105 is highlighted within the dotted square close to the C-terminus. In the right view, ND1 is not shown. The residue E105 is located close to the matrix side of ND3 just before the C-terminal tail in proximity to ND6 and ND1. (D) Detailed view of E105 indicates that there are no potential interactors in its proximity on ND3 (orange), ND1 (gray), or ND6 (turquoise). (E) Superimposed SWISS-MODEL predictions of the canonical ND3 sequence (gray) and ND3.E105G (plumb) are structurally identical.

To further characterize the muscle mitochondria carrying the m.10372A>G mutation, blue native polyacrylamide gel electrophoresis (BN-PAGE) of mitochondria isolated from the first biopsy was conducted. No deviation in the quantity or size of CI in the patient was seen compared with controls, indicating that the mutation in *MT-ND3* does not affect assembly of CI (figure 4A).

### Population Frequency and In Silico Analyses

The mutation was not found in 50,175 GenBank sequences according to MitoMap^24^ and not in 2,704 sequences in the Human Mitochondrial Genome Database (mtDB).^25^ The mutation was predicted to be pathogenic by the prediction tools PolyPhen2 (HumDiv: score 1.00, HumVar: score 0.99), SIFT (score: 0), and Combined Annotation Dependent Depletion (CADD, score 24.0), MutPred2 (altered ordered interface, *p* value = 0.003), PROVEAN (score: −6.767), and Rhapsody (score: 0.68). The residue is also highly conserved across different species down to the CI core subunit Nqo1 in *Thermus thermophilus* (figure 4B), and all 61 mammals in the mtSNP Database mtSAP^26^ had glutamate in the corresponding position (not shown). The glutamate residue at position 105 is located close to the C-terminal tail of ND3 that faces the mitochondrial matrix (figure 4C). In a human CI structure obtained by cryogenic electron microscopy (PDB:5XTC),^27^ the negatively charged glutamate does not establish ionic contacts with any of the predominantly hydrophobic residues in its proximity (figure 4D). Furthermore, modeling of mutant ND3 with SWISS-MODEL^28^ did not cause structural changes to the protein compared with the canonical ND3 sequence (figure 4E), indicated by an root-mean-square deviation score of 0.001. This suggests that the mutation does not affect the stability of CI, but might rather have functional consequences.

### Investigations of a Second Muscle Biopsy and Cultured Myoblasts Established From the Biopsy

**Figure 5:**
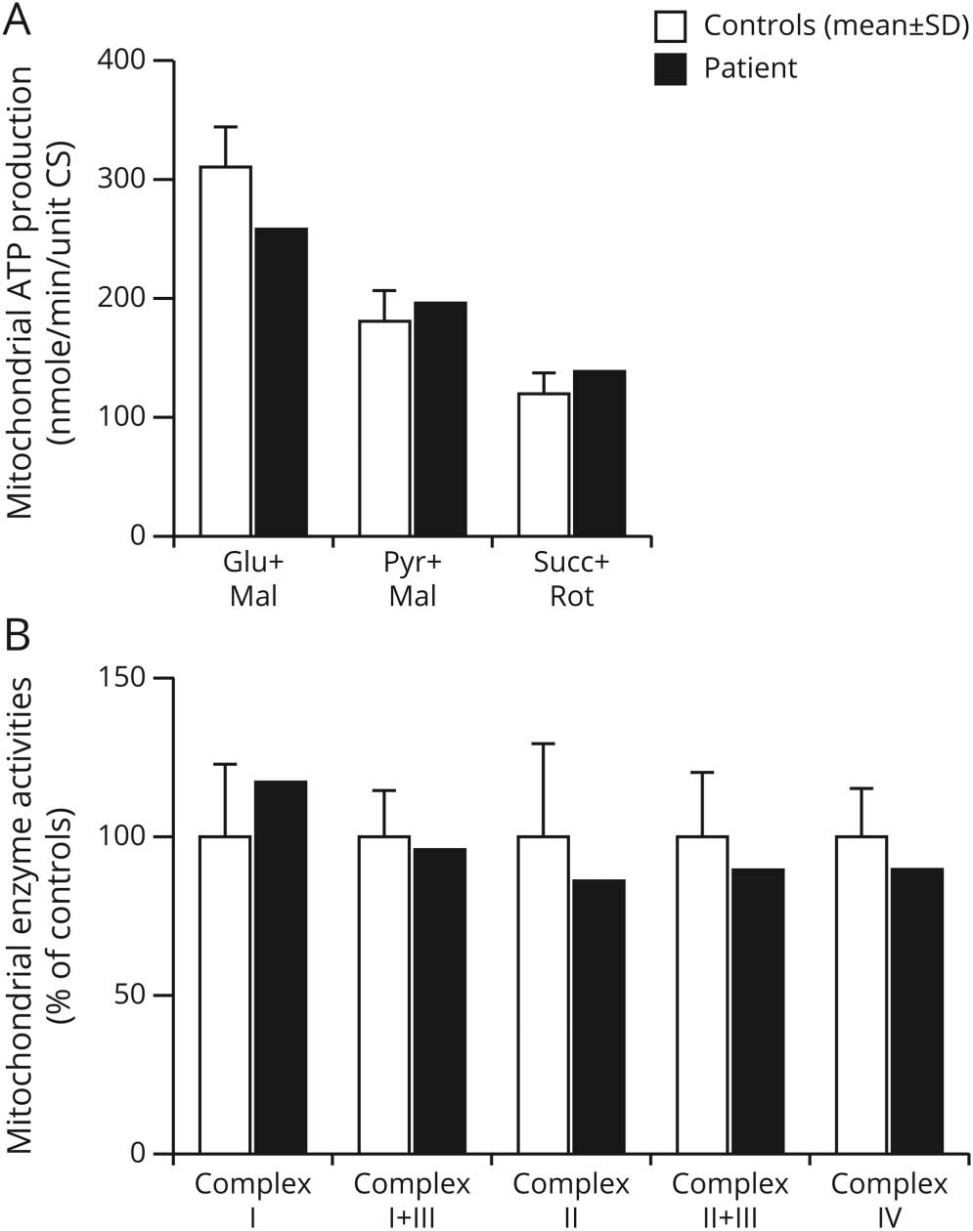
Biochemical Investigation of Mitochondria From Cultured Myoblasts Shows Normal Function (A) Mitochondrial ATP production is normal with the substrates involving CI, glutamate + malate, and pyruvate + malate compared with 8 healthy age-matched controls. (B) All complexes in the respiratory chain show normal activities compared with 6 healthy age-matched controls.

To establish a cell type carrying the mutation for further experiments, the patient was asked to undergo another muscle biopsy in 2016 when she was aged 55 years. The second muscle biopsy confirmed the morphologic (not shown) and biochemical analysis (figure 1, A and B) as well as the high mutation levels in muscle tissue (figure 3C). Cultured myoblasts derived from the same biopsy, however, did not carry the m.10372A>G mutation (figure 3D). To ensure that the cells in the myoblast culture from the patient were myogenic cells, immunocytochemistry with the myogenic marker desmin was performed (figure 3E). Primary fibroblasts from the patient were used as control cells for the staining (figure 3F). Sanger sequencing of the complete mtDNA from the cultured myoblasts showed the same sequence of m.1-16569 as for the former sequenced muscle DNA, except for the position m.10372. The fact that mtDNA from cultured myoblasts vs muscle tissue from the same biopsy only differed in 1 position gave us the opportunity to investigate the functional consequences of this novel mutation. We therefore performed mitochondrial biochemical investigations of the myoblast cells. Analysis of the patient's myoblasts showed normal mitochondrial ATP production (figure 5A) and RC activity (figure 5B) compared with control myoblasts. This indicates that the previously identified CI deficiency in muscle tissue from the same biopsy was caused by the m.10372A>G mutation.

### Whole-Genome Sequencing Identified High Heteroplasmic Levels of the m.10372A>G but No Other Mutation Explaining the Disease

The m.10372A>G mutation had been identified before the era of massive parallel sequencing. A whole-genome sequencing (WGS) analysis of DNA from muscle targeting all disease-associated genes in Online Mendelian Inheritance in Man and all genes in Human MitoCarta 2.0 was thus performed later. This was performed to exclude additional mutations in nuclear genes that could potentially contribute to the CI deficiency or the clinical symptoms of the patient. As expected, the WGS analysis detected the former identified *MT-ND3* mutation at a high heteroplasmic level, but no other disease-causing mutation in any known nuclear disease gene was found that could explain the phenotype.

## Discussion

Here, we have identified a novel heteroplasmic mutation in *MT-ND3*, m.10372A>G (p.Glu105Gly), coding for a subunit in CI of the RC, in a Caucasian woman presenting with sensorimotor axonal polyneuropathy as an initial symptom at age 41 years.

To be considered pathogenic, mtDNA-encoded CI mutations must fulfill a number of accepted criteria in the CI mutation pathogenicity scoring system.^29^ This system is based on parameters such as biochemical defect in multiple and affected tissues, functional studies in, e.g., single-fiber and/or cybrid studies, the number of cases reported, heteroplasmy level of the variant, segregation with disease in the family, and finally conservation scores of the amino acid position. In our case, CI deficiency of the mitochondrial RC, numerous RRF, and paracrystalline inclusions were shown in muscle tissue, from 2 different biopsies, carrying the mutation. Single-fiber and/or cybrid studies were not possible to perform in this case because only 1 COX-negative fiber was found in the muscle. However, the fact that the mutation was lost in myoblasts from the patient provided a different opportunity for functional studies. Measurement of mitochondrial ATP production and RC activity in mitochondria isolated from cultured myoblasts, not carrying the mutation, was completely normal compared with controls. This confirms that the biochemical defect segregates with the mutation in the different investigated tissues from the patient, strengthening the pathogenicity of the mutation. Last-cycle hot PCR analysis showed the mutation to be heteroplasmic at a high level in muscle (97%), low level in cultured fibroblasts (3%), and not detectable in blood, urinary epithelial cells, or cultured myoblasts. Previous studies have shown that both pathogenic point mutations and single, large-scale mtDNA deletions, present in high heteroplasmy levels in patient skeletal muscle, have been absent in the patients' cultured myoblasts, suggesting that the mutations are not present or are at very low levels in muscle satellite cells.^30–32^ Levels of mutant mtDNA in blood and fibroblasts in these cases were also absent or at extremely low levels, comparable to observations made in the patient described here. However, a study of several cases of sporadic mtDNA deletions detected a similar level of deleted mtDNA in both mature muscle and muscle satellite cells.^33^ In the majority of those patients, the deletions were shown to be completely lost in the transition from satellite cells to myoblasts, which also is a possible explanation for the loss of the mutation in our case. Muscle biopsies from relatives from the patient would have been required for segregation analysis, but unfortunately, this was not possible. BN-PAGE of mitochondria isolated from muscle showed normal size and quantity of CI, indicating that the mutation does not affect assembly of CI. Other pathogenic mutations in *MT-ND3* such as m.10158T>C and m.10191T>C have been shown to result in moderately reduced amounts or fully assembled CI and a decrease in enzyme activity.^17^ Those mutations are located in the loop connecting the first and second transmembrane helix of ND3, which is involved in the Active/Deactive transition of the enzyme.^34^ The proposed reason for CI dysfunction caused by those mutations has thus been impaired catalytic turnover of CI rather than structural destabilization.^35^ It has also been shown that locking of this loop of ND3 in *Yarrowia lipolytica* disengages proton pumping.^36^ Our modeling of the mutation m.10372A>G (p.(Glu105Gly)) positioned close to the C-terminal tail of ND3 in the mitochondrial matrix, indicates that Gly105 does not alter the protein structure compared with the canonical Glu105. Yet, the molecular relevance of this single negatively charged glutamate residue is unknown. Acidic residues like glutamate contribute to proton pumping^37^ or hydration of the complex.^38^ Similarly, one could argue that Glu105 is involved in the rearrangement of ND3 during CI energy conversion and might thus be of relevance to the fourth proton pathway in the membrane arm, attributed to a region close to ND1, ND6, and ND3.^39^ This could explain why p.Glu105Gly does not affect CI stability but rather has functional consequences through the loss of negative charge and gain of hydrophobicity.

The mutation has not previously been reported in MitoMap^24^ or the mtDB^25^ and is predicted to be pathogenic, using PolyPhen2, SIFT, and CADD. The extent of conservation was assessed with the mtSNP Database mtSAP,^26^ which showed that there was no variation at this amino acid position in any of the 61 mammals in the database. There was, however, variation in 2 of 4 amino acid residues surrounding the amino acid residue. Application of the pathogenicity scoring system placed the mutation in the group of probably pathogenic, with a score of 29 of possible 40 points, where a score of 30 or more is required for the category pathogenic. Segregation analysis in the family would have given the mutation another 3 points with a total score of 32 points. WGS did not identify any other variant that could explain the CI deficiency or the clinical symptoms of the patient, further supporting pathogenicity.

In summary, we describe a novel mtDNA mutation, m.10372A>G in *MT-ND3*, with a striking tissue-specific distribution. The mutation is present at high levels in muscle tissue but is lost in cultured myoblasts, providing an unusual opportunity to investigate its functional consequences. Our data strongly suggest that the identified mutation is pathogenic and responsible for the CI deficiency and clinical symptoms of the patient. This extends the clinical phenotypes of *MT-ND3* mutations as well as the known genetic aetiologies of mitochondrial polyneuropathy, highlighting the importance of mitochondrial investigation, including muscle biopsy, in patients presenting with seemingly idiopathic polyneuropathy, especially with coexisting muscle weakness.

## Glossary

ATP: adenosine triphosphate
BN-PAGE: blue native polyacrylamide gel electrophoresis
CADD: Combined Annotation-Dependent Depletion
CI: complex I
mtDB: Mitochondrial Genome Database
mtDNA: mitochondrial DNA
OM: oligomycin
RC: respiratory chain
RRF: ragged red fiber
WGS: whole-genome sequencing
